# Determinants of Cone Volume During Large Loop Excision of the Transformation Zone (LLETZ) and Their Association with Specimen Fragmentation and Excisional Margin—A Cross-Sectional Study

**DOI:** 10.3390/jcm15187139

**Published:** 2026-09-14

**Authors:** Dominika Trojnarska, Krzysztof Górnisiewicz, Justyna Kot, Marzena Wielgus, Iwona Gawron, Robert Jach

**Affiliations:** 1 Department of Maternal and Child Health, Institute of Nursing and Midwifery, Faculty of Health Sciences, Jagiellonian University Medical College, Kopernika 25, 31-501 Kraków, Poland; 2 Clinical Department of Gynecological Endocrinology and Gynecological Oncology, University Hospital in Kraków, Kopernika 23, 31-501 Kraków, Poland; 3 Chair of Gynecology and Obstetrics, Faculty of Medicine, Jagiellonian University Medical College, Kopernika 23, 31-501 Kraków, Poland

**Keywords:** conization, electrosurgery, margins of excision, cervical dysplasia, specimen fragmentation, cone volume

## Abstract

**Objective:** Given the variability in conization cone volume, specimen fragmentation, and histopathological excisional margin status, even in optimally performed procedures, the study aimed to assess the factors affecting these characteristics. **Methods:** This cross-sectional study based on retrospectively collected data, included 179 women who underwent large-loop excision of the transformation zone (LLETZ). The impact of age, menopausal status, parity, high-risk human papillomavirus (hr-HPV) status, transformation zone (TZ) type, conization indications, and histopathological characteristics on cone volume, fragmentation, and margin status was assessed. **Results:** Cone volume was positively associated with increased parity and was significantly larger in women with resection margin involvement and complete lesion excision compared to those with unspecified margins. No significant correlations were found between cone volume and age, menopausal status, hr-HPV status, TZ type, indications for LLETZ, or specimen fragmentation. Specimen fragmentation demonstrated no correlations with parity, menopausal status, LLETZ indications, or hr-HPV status, but it was more prevalent among women aged 31–35 years. Absence of fragmentation was linked to clear resection margins, while fragmentation correlated with margin involvement. Complete excision was more frequent in women with high-grade squamous intraepithelial lesions (HSIL) cytology, although lesions extended to the specimen margin primarily in specimens with biopsy-confirmed HSIL cervical intraepithelial neoplasia (CIN) 3. None of the evaluated factors were independent predictors of complete lesion excision or margin involvement. **Conclusions:** Cone volume, fragmentation, and margin status are clinically relevant LLETZ outcomes, although no independent predictors were identified after adjustment. Larger prospective multicenter studies are needed to determine how oncologic adequacy can be balanced with preservation of cervical function.

## 1. Introduction

Cervical cancer is the fourth leading cause of cancer among women and is responsible for approximately 350,000 annual deaths worldwide [1]. Timely diagnosis is crucial given the lengthy precancerous phase, which is typified by cervical intraepithelial neoplasia grades 2 or 3 (CIN 2–3). Both excisional and ablative procedures are available for the treatment of cervical precancerous lesions [2,3]. Techniques such as large-loop excision of the transformation zone (LLETZ), loop electrosurgical excision procedure (LEEP), laser excision, cold knife conization, laser vaporization, and cryotherapy can be used to remove these lesions effectively and achieve disease eradication. No single conservative surgical technique is universally recognized as superior for the treatment and eradication of squamous intraepithelial lesions (SIL) [4]. However, in patients with high-grade SIL (HSIL) with CIN 2–3, excisional methods are preferred for histopathological assessment and detection of potential invasive disease [4]. Regardless of the chosen approach, the current standard is the tissue-sparing excision of the lesion with clear margins under colposcopic guidance, in most cases including the transformation zone [5]. Selection of the excisional technique should be individualized based on relevant clinical characteristics [6]. LLETZ should be regarded as both a therapeutic and diagnostic procedure. In addition to treating lesions confined to the ectocervix or those whose upper margin is visible within the endocervical canal, LLETZ provides advantages comparable to those of conization. It enables excision of a cone-shaped specimen for histopathological evaluation, thereby facilitating definitive diagnosis [6,7,8]. The type and extent of transformation zone excision are determined by the transformation zone type, lesion extent, and suspected endocervical involvement. In selected diagnostically uncertain cases, LLETZ can also be performed for diagnostic purposes according to local clinical practice; this does not imply that smaller excisions are appropriate for extensive lesions or type 3 transformation zones. Its drawbacks relate to the difficulty in performing a histological assessment of the margins due to thermal damage.

The risk of CIN 2–3 recurrence is associated with several factors, including patient age, severity of the initial lesion, and excisional margin status [9,10,11]. Regarding surgical variables, cold-knife conization appears to carry the highest risk of prematurity, with the risk further increasing as excisional specimen depth increases, suggesting that surgeons should avoid this technique when feasible and preserve non-pathologic cervical tissue as much as possible [12,13]. Complete removal of the lesion while preserving as much healthy cervical tissue as possible is crucial. This approach minimizes the risk of disease recurrence and reduces the likelihood of cervical insufficiency, which is particularly important for women planning future reproduction, as all excisional procedures are associated with similar pregnancy-related morbidities [14]. Excisional treatment for CIN prior to pregnancy has been associated with an increased risk of preterm birth, with reported incidences ranging from 11.2% in a meta-analysis of observational studies to 25.3% in a recent large-scale registry-based study from Japan, compared with non-treated women [15,16]. Given the established positive correlation between excised specimen size and the risk of obstetric complications, women with CIN 3 are increasingly managed using more conservative treatment approaches. However, this trend may potentially be associated with an increased risk of CIN recurrence and cervical carcinoma [17].

In women desiring future fertility, preservation of healthy cervical tissue should be prioritized whenever feasible. However, this principle also applies to women without reproductive intentions, as excision of larger tissue volumes, even though it may improve the likelihood of complete lesion removal, has been associated with an increased risk of postoperative complications, including bleeding and cervical stenosis [3].

This study investigated factors potentially influencing LLETZ cone volume and examined its association with specimen fragmentation and excisional margin status. Specifically, we sought to determine whether larger cone volumes correlated with improved clinical outcomes, such as CIN-free margins, or whether additional factors—including patient characteristics, procedural techniques, and underlying pathology—contributed to variations in cone size. We hypothesized that LLETZ performed without prior histological confirmation may be approached more conservatively because of its partly diagnostic purpose, potentially resulting in a smaller excised specimen. Conversely, histologically confirmed high-grade squamous intraepithelial lesion (HSIL), particularly when accompanied by hr-HPV positivity or high-grade cytology, may increase concern regarding clinically significant disease and prompt a more extensive excision. Because direct evidence linking these preprocedural findings to cone volume is limited, their associations with cone volume were evaluated as exploratory rather than confirmatory objectives.

The clinical novelty of this study lies in its integrated assessment of LLETZ specimen volume, potential factors associated with fragmentation, and the effects of both specimen volume and fragmentation on resection margin status.

## 2. Materials and Methods

This cross-sectional study, approved by the Jagiellonian University Medical College Research Ethics Committee, approval number 118.0043.1.165.2024, recruited women who had undergone LLETZ procedures due to squamous intraepithelial lesions (SIL) between January 2022 and July 2024 in the Clinical Department of Gynecological Endocrinology and Gynecological Oncology at the University Hospital in Kraków.

The study group comprised individuals aged 18 years and older who underwent LLETZ. Women under 18 years of age and those with incomplete medical records that precluded verification of the indication for treatment were excluded from the study. All procedures related to data collection were conducted in accordance with the Declaration of Helsinki, and written informed consent was obtained from each participant. The study population constituted a convenience sample comprising all eligible women who underwent LLETZ at our institution between January 2022 and July 2024.

The clinical data, such as patient age, menopausal status, parity, cervical cytology results, hr-HPV status, colposcopy results, including TZ classification according to the IFCPC nomenclature [18], histopathological results, and HPV vaccination status, were collected from the electronic medical records.

Due to the retrospective nature of the study and limitations of the available operative and pathology records, it was not possible to distinguish between intentional (planned) and technical (unintentional) specimen fragmentation. Margin status was classified as clear when lesions were completely excised without involvement of surgical margins, involved when lesions extended to the margin, and uncertain when assessment was limited by specimen fragmentation and/or thermal artifact. Cases were categorized as unknown when margin status was not reported or could not be retrieved from the pathology record. We assessed whether fragmentation was associated with margin involvement or indeterminate margin assessment and whether specimen volume was related to the completeness of excision. Additionally, we explored factors potentially associated with fragmentation and margin status, including demographic and clinical variables. Given the retrospective design and multifactorial nature of the analyses, these secondary comparisons were considered exploratory and hypothesis-generating rather than confirmatory. We further explored whether younger, nulliparous, and premenopausal women tended to undergo smaller excisions with lower fragmentation rates, potentially reflecting a more conservative surgical approach aimed at preserving cervical integrity in women of reproductive age. Previous cervical cytology, preceding biopsy findings, and hr-HPV status were included as exploratory clinical variables because these results were available to the treating clinician before LLETZ and could potentially influence the planned extent of excision. We therefore examined whether differences in the preprocedural evidence supporting the indication for LLETZ were associated with cone volume. These analyses were hypothesis-generating, as these variables have not been previously established as determinants of cone volume.

The obtained data were entered into Microsoft® Excel for Mac, version 16.109.3 (Microsoft Corporation, Redmond, WA, USA), for statistical analysis. Quantitative variables were summarized using descriptive statistics, including the mean, standard deviation, median, quartiles, minimum, and maximum. Categorical variables were summarized as absolute frequencies and percentages. Categorical variables were compared between groups using the chi-square test (with Yates’ continuity correction for 2 × 2 contingency tables) or Fisher’s exact test when the assumptions of the chi-square test regarding expected cell counts were not met. Quantitative variables were compared between two groups using the Mann–Whitney U test. Comparisons involving three or more groups were performed using the Kruskal–Wallis test, followed, when statistically significant, by Dunn’s post hoc test with Holm’s correction for multiple comparisons. Multivariable linear regression was used to assess the effects of potential predictors on quantitative outcomes. Results are reported as regression coefficients with 95% confidence intervals (CIs). Multivariable logistic regression was used to assess the effects of potential predictors on dichotomous outcomes. Results are reported as odds ratios (ORs) with 95% CIs. Statistical significance was set at a two-sided *p*-value of <0.05. All statistical analyses were performed using R software, version 4.4.1 (R Foundation for Statistical Computing, Vienna, Austria).

The procedures of colposcopy and LLETZ were performed according to international standards [19,20]. The identified abnormalities were classified in accordance with IFCPC 2011 nomenclature [18]. All LLETZ procedures were conducted by a specialist in obstetrics and gynecology or by a trainee physician under direct specialist supervision, following the institution’s standard operating procedures. The tissue specimen was evaluated within a dedicated pathology department, where the personnel collaborated closely to ensure the utmost consistency in the execution of procedures and the assessment of results.

## 3. Results

A total of 186 LLETZ procedures were identified. After excluding 7 cases with incomplete medical records that precluded verification of the indication for treatment, data from 179 women were included in the analysis.

The characteristics of the study group are presented in Table 1.

The age of the participants ranged from 21 to 81 years, with a mean of 39.32 years (SD = 12.24). The majority of women (86.59%) were premenopausal. Regarding parity, 39.11% were multiparous, 21.23% were primiparous, and 37.43% were nulliparous. All women younger than 25 years with histologically confirmed HSIL were counselled regarding conservative management, including close surveillance and repeat colposcopic assessment and biopsy after 3 months, as well as the potential benefits and risks of conservative and surgical management. Following shared decision-making, the patients included in this study chose LLETZ because of concerns about disease persistence or progression.

HPV vaccination status was largely negative, with 83.24% reporting no vaccination. Up to June 2025, the costs of HPV testing were not reimbursed by the Polish social security system. Consequently, HPV testing was not conducted in 51.96% of the study population, while 18.44% of the participants tested positive for HPV types 16/18, and 10.61% tested positive for hr-HPV non-16/18 types.

Cytological examination revealed HSIL in 43.02% of women, low-grade squamous intraepithelial lesion (LSIL) in 26.82%, and ASC-US in 9.50%. More severe findings included adenocarcinoma in 1.12% and squamous cell carcinoma (SCC) in 1.68% of women. All women with NILM were hr-HPV positive.

Regarding the TZ type, 43.02% had a type 1 TZ, and 26.82% had a type 3 TZ, with TZ data unavailable for 16.76% of participants, as seen in the statistical analysis of specimen-dependent characteristics.

The median cone volume was 3125.00 mm^3^ (IQR: 2059.50–5480.00 mm^3^), while the mean was 4233.44 ± 3334.80 mm^3^. Specimen fragmentation occurred in 90 cases (50.28%), while clear resection margins were obtained in 107 patients (61.1%). Consequently, margin involvement was identified in 109 patients (60.89%).

Among 179 patients undergoing LLETZ, 125 (69.83%) had histologically confirmed high-grade disease: CIN3 in 59 cases, CIN2 in 64, and AIS in two. A further 23 patients (12.85%) had LSIL on biopsy or cervical curettage; in 18 of these cases, additional concerns were documented, including hr-HPV positivity, a type 3 transformation zone, abnormal endocervical findings, or discordant ASC-H or AGC cytology. In the remaining 31 patients (17.32%), the indication was based primarily on cytological findings: HSIL in 17 cases, ASC-H in one, suspected adenocarcinoma in two, LSIL in nine, and ASC-US in two. hr-HPV positivity and/or a type 3 transformation zone contributed to decision-making in several of these cases. Overall, indications were frequently multifactorial and reflected histological or cytological abnormalities combined with virological findings, transformation zone visibility, and endocervical assessment. For statistical analyses, these detailed indications were consolidated into broader categories.

We analyzed the volume of the conization cone, specimen fragmentation, and margin status in relation to the indications for surgical treatment (Table 2).

The largest specimen volume was observed in women with LSIL confirmed by biopsy, whereas the smallest specimen was associated with LSIL detected via cytology. Histopathologically confirmed HSIL (CIN2) was associated with the highest prevalence of cone fragmentation, whereas single-piece specimens were predominantly obtained from women with cytological suspicion of LSIL. No significant differences were found between specimen volume or fragmentation and surgical indications.

A statistically significant correlation was observed between the indications for conization and margin status (*p* = 0.016). The highest percentage of completely excised lesions with clear margins was observed in patients with HSIL diagnosed by cytology (66.67%).

No significant associations were found between age, menopausal status, hr-HPV status, or TZ type and the specimen volume. A statistically significant correlation was observed between parity and specimen volume (*p* < 0.001), which was significantly greater in women who had given birth to three or more children than in those who had given birth to two, one, or no children. Women who had two childbirths had significantly larger specimen volumes compared to nulliparous women (Table 3).

The specimen volume was significantly greater in patients with lesion involvement at the resection margin and in completely excised specimens (*p* = 0.021) than in those with indeterminate margins. No statistically significant effect of fragmentation on specimen volume was observed (Table 4).

No significant associations were found between cone fragmentation and menopausal status, parity, or hr-HPV status. Statistically significant differences were observed concerning age (*p* = 0.037) and TZ type (*p* = 0.001). The highest rate of specimen fragmentation was observed in women aged 31–35 years and in patients with TZ1. Fragmentation of the specimen was found to have a significant impact on margin status. The rate of complete excision was higher in women without fragmentation (*p* < 0.001). Detailed results are presented in Table 5.

In the multivariable linear regression analysis, surgical indication, hr-HPV status, and TZ type were not independently associated with specimen volume (Table 6). In the multivariable logistic regression analyses, none of these variables were significant independent predictors of specimen fragmentation (Table 7), complete excision, or lesion involvement at the resection margin (Table 8).

## 4. Discussion

The study found no significant differences between surgical indications and specimen volume or fragmentation. However, a significant association was identified between the indications for conization and resection margin status, with the highest percentage of completely excised lesions observed in women diagnosed with HSIL based on cytological suspicion and without prior biopsy. Complete excision of cervical dysplastic lesions within the excised specimen enables the procedure to serve both diagnostic and therapeutic purposes [6]. In contrast, involved surgical margins were most frequently found in specimens obtained from patients with HSIL, specifically CIN3, which could be explained by prior colposcopy-guided biopsy excluding invasive disease and consequently favoring a less extensive excisional procedure.

The management of women with a TZ 3 is particularly reliant on cytology results and biopsy findings obtained during colposcopy. In cases of high-grade squamous referral cytology, a diagnostic type 3 excision is an acceptable option for accessing the endocervical canal for potential unrecognized disease [21]. Type 3 excision is associated with a higher rate of clear margins, potentially due to the greater specimen length enabling complete endocervical excision [22]. We hypothesized that the volume of the conization specimen would be influenced not only by TZ type but also by women’s age, menopausal status, parity, and hr-HPV status. However, contrary to our expectations, the only statistically significant correlation was observed with parity, as cone volume was greater in women who had given birth at least twice. Previous studies have reported similar findings, demonstrating that the largest transverse diameter of the LLETZ specimen was significantly greater in patients who did not plan to conceive [23]. Positive endocervical margin involvement was associated with older age, possibly owing to inward migration of the squamocolumnar junction and endocervical lesion extension, whereas ectocervical margin involvement was associated with younger age, potentially reflecting fertility-preserving excision, ectropion, and externalization of the squamocolumnar junction [24]. Although significant cervical regeneration within six months after LLETZ has been reported—suggesting substantial, though incomplete, volumetric regeneration—previous studies indicate that greater thickness and total volume of the excised transformation zone are associated with an increased risk of preterm labor [25]. Interestingly, women with CIN have been demonstrated to have a higher baseline risk of preterm birth compared to the general population, even without treatment [26]. However, local treatment for CIN has significantly increased the risk of preterm birth compared to having CIN alone, as indicated by previous research. The most critical risk factor for preterm delivery has been the need for repeated intervention, particularly at a younger age [27]. Moreover, a history of cervical excisional treatment has been linked to alterations in the cervical microbiota composition in pregnant women with preterm premature rupture of membranes [28,29], and conization has been associated with an increased risk of clinical chorioamnionitis, particularly among women who experience preterm birth [30].

Histopathological reports should always document specimen dimensions and assess resection margins for the presence of intraepithelial or invasive disease [4], as positive endocervical margins are the main risk factor for 5-year recurrence of HSIL [11]. In our study sample, clear margins were achieved in the majority of cases. These findings are broadly consistent with previous reports; however, the rate of clear margins was more favorable in our study than previously described [22]. Literature has reported that women treated for HSIL remain at an increased risk of recurrent disease and cervical cancer for up to 25 years. Key factors associated with a higher risk include lesion size and grade, advanced age, treatment modality, incomplete excision, and persistent infection with hr-HPV [10,21]. It is reasonable to assume that a larger specimen volume would increase the likelihood of complete lesion excision. Indeed, the specimen volume was significantly greater in women with clear resection margins compared to those with indeterminate margins. However, this association was also observed in individuals whose lesions extended to the resection margin. The possibility of false-positive margin assessment due to heat-induced artifacts, which may misleadingly indicate incomplete excision, should be carefully considered [31]. While effective, LLETZ introduces a degree of thermal injury to the excised specimen, compromising the ability to accurately assess resection margins. In follow-up care after treatment for SIL, independent of resection margins, a combined examination using both hr-HPV testing and cytology, or hr-HPV testing alone, should be performed [32].

When utilizing excisional techniques for SIL treatment, every effort should be made to excise the lesion as a single, intact specimen [4]. However, it can be assumed that fragmentation—intentional versus technical—could result in larger specimen volumes. Nevertheless, our findings demonstrated no statistically significant effect of fragmentation on specimen volume. Importantly, we confirmed that the rate of complete excision with clear margins was greater in patients without fragmentation, whereas lesion involvement at the resection margin was more frequently observed when the specimen was fragmented. Fragmentation may impair histopathological interpretability by complicating specimen orientation, margin assessment, and evaluation of lesion extent [4]. Indications for LLETZ, hr-HPV status, and TZ type could theoretically influence the conization cone volume, fragmentation, and resection margins. However, our analysis demonstrated that none of these variables were significant independent predictors of these characteristics.

Notably, fewer than 4% of women in our study population had received HPV vaccination. Although HPV vaccination is well established as a safe and effective strategy for preventing HPV-related diseases, growing evidence also supports its potential therapeutic benefit, particularly in reducing the risk of recurrent cervical dysplasia following treatment [33,34]. However, the implementation of HPV vaccination in clinical practice remains suboptimal. A recent study demonstrated that although Polish obstetrician–gynecologists generally hold positive attitudes toward HPV vaccination, substantial inconsistencies persist regarding dosing regimens, timing of administration, and recommendations for vaccination after HSIL treatment [35]. Such variability may contribute to inequities in access to evidence-based prevention strategies. Therefore, strengthening the implementation of HPV vaccination, particularly among women undergoing treatment for HSIL, may represent an important opportunity to improve both the effectiveness and equity of cervical cancer prevention [36,37].

Several limitations of this study should be acknowledged. First, the retrospective single-center design inherently limits causal inference, and the observed associations should therefore be interpreted as exploratory and hypothesis-generating rather than causal. As a retrospective analysis, the study is susceptible to selection bias, information bias related to incomplete medical documentation, and unmeasured confounding. Importantly, factors that may substantially influence surgical outcomes—including surgeon intraoperative decision-making, lesion complexity, transformation zone characteristics, lesion size, colposcopic findings, technical difficulty of the procedure, and the intended diagnostic versus therapeutic nature of excision—were not systematically available and therefore could not be fully accounted for in the analyses. While a single-center protocol may limit external validity, thereby necessitating comparisons with multi-center or international datasets, there is currently a lack of data in the literature that would allow for a direct comparison to our original study. The conclusions derived from our research may serve to initiate such analyses.

A methodological limitation concerns the assessment of specimen volume. Ideally, excised tissue volume should be determined using Archimedes’ principle, which provides a more accurate volumetric estimation [3]. However, the retrospective nature of the study precluded the use of this approach. Instead, specimen volume was estimated using a simplified geometric approximation based on the multiplication of three measured dimensions, assuming a rectangular box shape, as applied in comparable studies [38]. Nevertheless, because LLETZ specimens are frequently irregular in shape, this method may introduce systematic measurement error. Additionally, tissue shrinkage associated with fixation and the lack of assessment of interobserver variability in pathological measurements may have further affected volume estimation and reproducibility. Despite the limitations, the calculated specimen volumes were comparable to those reported in previous studies [22].

Another potential source of variability relates to operator-dependent factors. Previous reports have suggested that operator characteristics may influence excision patterns, particularly regarding cone size in women with differing reproductive intentions [39]. Residual confounding cannot be excluded because potentially relevant factors, including lesion size, surgeon experience, and loop selection, were not assessed. These factors may have influenced the extent of excision, specimen fragmentation, and resection margin status; therefore, the corresponding findings should be interpreted with caution.

The absence of hr-HPV testing in more than half of the participants represents an additional limitation. As the testing has only been incorporated into the Polish cervical cancer screening program since July 2025, it was often financially inaccessible during the study period. Consequently, analyses involving hr-HPV status were restricted to complete-case evaluation and may be prone to bias due to missing data. The incomplete availability of hr-HPV data may have reduced the precision of estimates concerning the association between hr-HPV status and surgical outcomes.

Clinical heterogeneity within the study population should also be acknowledged, as indications for LLETZ included a spectrum of cytological, histopathological, and HPV-related abnormalities, potentially reflecting different baseline risks and treatment pathways. Furthermore, the relatively limited sample size may have reduced statistical power, potentially contributing to the absence of statistically significant independent predictors. The analyses of previous cytology, biopsy findings, and hr-HPV status should be interpreted as exploratory. Although these variables may influence the clinician’s preprocedural assessment and intended extent of excision, the study did not directly measure procedural decision-making, and the observed associations cannot establish that these findings determined cone volume.

Despite these limitations, the study has several strengths. It provides a comprehensive evaluation of multiple carefully collected clinical and histopathological variables relevant to LLETZ specimen characteristics. Furthermore, the study was conducted in a specialized center dedicated to the treatment of women of reproductive age, enhancing the clinical relevance of the findings in this patient population.

## 5. Conclusions

Cone volume, fragmentation, and margin status are clinically relevant characteristics of LLETZ specimens; however, their determinants are multifactorial and incompletely captured in retrospective datasets. In this single-center study population, no definitive independent predictors were identified after adjustment, which may reflect limited statistical power, clinical heterogeneity, and unmeasured confounding. Larger prospective multicenter studies incorporating lesion size, colposcopic findings, transformation zone type, surgical technique, operator experience, hr-HPV status, recurrence, and reproductive outcomes are needed to define evidence-based strategies for balancing oncologic adequacy with preservation of cervical function.

## Figures and Tables

**Table 1:** Characteristics of the study group.

Characteristic		N = 179
Age at LLETZ [years]	Mean (SD)	39.32 (12.24)
	Median (quartile)	36 (31–45)
	Range	21–81
Menopausal status	Premenopausal	155 (86.59%)
	Postmenopausal	24 (13.41%)
Parity	Nullipara	67 (37.43%)
	Primipara	38 (21.23%)
	Multipara	70 (39.11%)
	Unknown	4 (2.23%)
HPV vaccination	No	149 (83.24%)
	Yes	7 (3.91%)
	Unknown	23 (12.85%)
Cytology	NILM	6 (3.35%)
	AGC	3 (1.68%)
	ASC-US	17 (9.50%)
	ASC-H	14 (7.82%)
	LSIL	48 (26.82%)
	HSIL	77 (43.02%)
	Adenocarcinoma	2 (1.12%)
	SCC	3 (1.68%)
	Unknown	9 (5.03%)
HPV status	Not tested	93 (51.96%)
	Negative	6 (3.35%)
	Non-16/18	19 (10.61%)
	16/18	33 (18.44%)
	16/18 and non-16/18	15 (8.38%)
	Unknown	13 (7.26%)
Transformation zone	1	77 (43.02%)
	2	24 (13.41%)
	3	48 (26.82%)
	Unknown	30 (16.76%)

LLETZ—large-loop excision of the transformation zone; SD—standard deviation; HPV—human papillomavirus; NILM—negative for intraepithelial lesion or malignancy; AGC—atypical glandular cells; ASC-US—atypical squamous cells of undetermined significance; ASC-H—atypical squamous cells—cannot exclude HSIL; LSIL—low-grade squamous intraepithelial lesion; HSIL—high-grade squamous intraepithelial lesion; SCC—squamous cell carcinoma.

**Table 2:** Conization specimen volume, fragmentation, and margin status according to surgical treatment indications.

Indication	Volume, Mean (SD) mm^3^	Volume, Median (IQR) [mm^3^]	Fragmentation n (%)	Clear margins n (%)
HSIL (CIN3) histology	3976.37 (2807.31)	2975 (2060–5000)	30 (50.85)	38 (64.41)
HSIL (CIN2) histology	4097.30 (2815.18)	3137.5 (2250–5470)	38 (59.38)	39 (60.94)
LSIL histology	5832.52 (5870.31)	3200 (2270–7312)	7 (30.43)	12 (52.17)
HSIL cytology	3835.28 (2495.67)	3384 (1940–5048)	8 (44.44)	12 (66.67)
LSIL cytology	3951.82 (2676.02)	2700 (1638–6825)	3 (27.27)	6 (54.55)
*p*	0.926 ^a^		0.083 ^b^	0.016 ^c^*

*p*—categorical variables: ^a^ Kruskal‐Wallis test; ^b^ Pearson’s chi-square test; ^c^ Fisher’s exact test; * Statistically significant (*p* ≤ 0.05); IQR—interquartile range.

**Table 3:** Factors associated with conization specimen volume.

Parameter	Group	Median Volume (IQR) mm^3^	*p*
Age	≤30	2700 (1785–4500)	0.149
	31–35	3035 (2240–5500)	
	36–45	3634 (2250–5625)	
	>45	2880 (2120–6687)	
Menopausal status	Premenopausal	3125 (2060–5240)	0.400
	Postmenopausal	3937.5 (2059–6038)	
Parity	0	2448 (1792–4306)	<0.001 *
	1	3182 (2198–4862)	
	2	3462.5 (2227–5404)	
	≥3	5653 (3064–9805)	
HPV	Unknown	3600 (2201–5767)	0.081
	Negative	1625 (1233–5448)	
	Positive	2880 (1833–4650)	
Transformation zone	1	2900 (2250–5100)	0.92
	2	3394 (2130–5525)	
	3	3600 (1815–6006)	

*p*—comparison of 2 groups: Mann‐Whitney test; comparison of more than 2 groups: Kruskal‐Wallis test + post hoc analysis (Dunn’s test); * Statistically significant (*p* ≤ 0.05); IQR—interquartile range; HPV—human papillomavirus.

**Table 4:** Conization specimen volume according to margin and fragmentation status.

Characteristic	Group	Median Volume (IQR) mm^3^	*p*
Margin status	Uncertain	2600 (1331–3600)	0.021 *
	Clear	3200 (2250–5460)	
	Involved	4600 (2261–7196)	
Fragmentation	No	2992 (2040–5000)	0.710
	Yes	3182 (2201–5675)	

*p*—comparison of 2 groups: Mann‐Whitney test; comparison of more than 2 groups: Kruskal‐Wallis test + post hoc analysis (Dunn’s test); * Statistically significant (*p* ≤ 0.05); IQR—interquartile range.

**Table 5:** Factors associated with conization specimen fragmentation and margin status.

Characteristic	Group	Fragmentation n (%)	Margin Status/Key Finding	*p*
Age	<30	19 (46.34)	—	0.037 *
	31–35	29 (64.44)	—	
	36–45	18 (36.0)	—	
	≥45	24 (55.81)	—	
Menopausal status	Premenopausal	76 (49.03)	—	0.530
	Postmenopausal	14 (58.33)	—	
Parity	0	33 (49.25)	—	0.200
	1	20 (52.63)	—	
	2	19 (43.18)	—	
	≥3	18 (69.23)	—	
HPV status	Unknown	56 (52.83)	—	0.249
	Negative	1 (16.67)	—	
	Positive	33 (49.25)	—	
Transformation zone	1	50 (64.94)	—	0.001 *
	2	11 (45.83)	—	
	3	15 (31.25)	—	
Fragmentation vs. margins	No	—	Clear: 68 (76.40%); Involved: 6 (6.74%)	<0.001 *
	Yes	—	Clear: 41 (45.56%); Involved: 17 (18.89%)	

*p*—Chi-square test or Fisher’s exact test; * Statistically significant (*p* ≤ 0.05); HPV—human papillomavirus.

**Table 6:** Determinants of conization specimen volume.

Parameter	Category	β Coefficient (95% CI)	*p*
Surgical indication	HSIL (CIN2) histology	225.4 (−971.6 to 1422.3)	0.713
	LSIL histology	1711.7 (−83.1 to 3506.6)	0.063
	HSIL cytology	−735.5 (−2694.9 to 1223.9)	0.463
	LSIL cytology	−364.5 (−2708.5 to 1979.4)	0.761
HPV status	Negative	−885.8 (−3791.4 to 2019.9)	0.551
	Non-16/18	−1399.9 (−3103.8 to 304.0)	0.109
	16/18	−890.3 (−2274.2 to 493.6)	0.209
Transformation zone	1	−774.8 (−2504.1 to 954.6)	0.381
	2	−1066.8 (−3041.1 to 907.4)	0.291
	3	−696.7 (−2310.0 to 916.5)	0.399

*p*—multiple linear regression; CI—confidence interval; HSIL—high-grade squamous intraepithelial lesion; LSIL—low-grade squamous intraepithelial lesion; HPV—human papillomavirus.

**Table 7:** Determinants of conization specimen fragmentation.

Parameter	Category	OR (95% CI)	*p*
Surgical indication	HSIL (CIN2) histology	1.50 (0.70–3.20)	0.295
	LSIL histology	0.83 (0.26–2.64)	0.752
	HSIL cytology	1.19 (0.35–4.00)	0.783
	LSIL cytology	0.76 (0.16–3.68)	0.737
HPV status	Negative	0.20 (0.02–2.06)	0.174
	Non-16/18	0.65 (0.22–1.91)	0.430
	16/18	0.61 (0.25–1.49)	0.276
Transformation zone	1	2.50 (0.85–7.39)	0.097
	2	1.00 (0.30–3.38)	1.000
	3	0.54 (0.19–1.50)	0.235

*p*—multiple logistic regression; OR—odds ratio; CI—confidence interval; HSIL—high-grade squamous intraepithelial lesion (cervical intraepithelial neoplasia grade 2 or 3); LSIL—low-grade squamous intraepithelial lesion; HPV—human papillomavirus.

**Table 8:** Determinants of conization specimen margin status.

Parameter	Category	Clear Margins OR (95% CI)	Involved Margins OR (95% CI)	*p* Clear/Involved Margins
Surgical indication	HSIL (CIN2) histology	0.95 (0.43–2.09)	0.42 (0.15–1.15)	0.89/0.091
	LSIL histology	0.95 (0.24–3.76)	—	0.938/—
	HSIL cytology	1.99 (0.35–11.28)	0.49 (0.08–2.98)	0.437/0.438
HPV status	Non-16/18	1.26 (0.41–3.92)	0.70 (0.13–3.65)	0.688/0.671
	16/18	1.14 (0.46–2.87)	1.15 (0.37–3.61)	0.774/0.805
Transformation zone	1	0.48 (0.14–1.67)	1.26 (0.20–8.08)	0.249/0.806
	2	0.72 (0.18–2.94)	1.58 (0.22–11.59)	0.645/0.652
	3	1.41 (0.40–4.96)	1.33 (0.21–8.65)	0.589/0.765

*p*—multiple logistic regression; OR—odds ratio; CI—confidence interval; HSIL—high-grade squamous intraepithelial lesion (cervical intraepithelial neoplasia grade 2 or 3); LSIL—low-grade squamous intraepithelial lesion; HPV—human papillomavirus.

## Data Availability

Dataset is available under: https://doi.org/10.34740/kaggle/ds/11684634.
